# Angle of Uterine Flexion and Adenomyosis

**DOI:** 10.3390/jcm11113214

**Published:** 2022-06-04

**Authors:** Anjeza Xholli, Umberto Scovazzi, Ambrogio Pietro Londero, Giulio Evangelisti, Elena Cavalli, Maria Giulia Schiaffino, Ilaria Vacca, Francesca Oppedisano, Mattia Francesco Ferraro, Giorgio Sirito, Filippo Molinari, Angelo Cagnacci

**Affiliations:** 1 Academic Unit of Obstetrics and Gynecology, IRCCS Ospedale Policlinico San Martino, 16132 Genova, Italy; anj160583@yahoo.it (A.X.); s3659175@studenti.unige.it (U.S.); evangelisti.giulio@gmail.com (G.E.); elenacavalli93@virgilio.it (E.C.); schiaffinomg@gmail.com (M.G.S.); ilodelpolmone@gmail.com (I.V.); foppedisano22@gmail.com (F.O.); mattiafrancescoferraro@yahoo.it (M.F.F.); giorgiosirito1893@gmail.it (G.S.); filippo.molinari2@gmail.com (F.M.); 2 Department of Neurology, Rehabilitation, Ophtalmology, Genetics, Maternal and Infant Health (DiNOGMI), 16132 Genova, Italy; ambrogio.londero@unige.it

**Keywords:** adenomyosis, dysmenorrhea, dyspareunia, chronic pelvic pain, retroverted uterus, transvaginal ultrasound

## Abstract

The aim of this study was to assess the prevalence of adenomyosis in symptomatic women in relation to the angle of flexion of the uterus. A total of 120 patients referring to our Chronic Pelvic Pain Center were prospectively enrolled. Each woman scored menstrual pain, intermenstrual pain, and dyspareunia on a 10 cm visual analogue scale and underwent a clinical examination and transvaginal ultrasound. MUSA criteria were used for the diagnosis of adenomyosis. The angle of flexion of the uterus on the cervix was categorized as <150° (75% of cases), between 150° and 210° (6.7% of cases) and >210° (18.3% of cases). Adenomyosis was diagnosed in 76/120 women (63.3%). In women with adenomyosis, the VAS of intermenstrual pain was higher than in women without adenomyosis (4.04 ± 3.79 vs. 2.57 ± 3.34; *p* < 0.034). The angle of uterine flexion >210° was more prevalent in women with than without adenomyosis (25.0% vs. 6.8%; *p* < 0.015). The odds ratio of suffering from adenomyosis markedly increased in the presence of an angle of uterine flexion >210° (OR 5.8 95% CI 1.19, 28.3; *p* > 0.029). The data indicate that the ultrasound-estimated angle of uterine flexion >210° is related to a higher prevalence of adenomyosis.

## 1. Introduction

Adenomyosis is the proliferation of endometrial glands and stroma within the myometrium. It is a common finding and in epidemiological data, its prevalence ranges from 10% to 40%. The symptoms affect mostly women of a reproductive age, with a higher prevalence in women over 40 [1,2,3]. It is unclear how endometrial tissue develops within the myometrium. Known risk factors are higher estrogen exposure, early menarche, polymenorrhea, parity, and previous uterine surgery. According to the most supported theory, ectopic endometrium directly infiltrates the myometrium, with a favorable environment resulting from physical and hormonal factors^2^. The diagnosis of adenomyosis can be reliably performed by histology, but in a conservative scenario, imaging techniques such as 2D transvaginal ultrasound or MRI can be used with a comparable high diagnostic sensitivity and specificity [4,5,6].

Typical symptoms of adenomyosis include abnormal uterine bleeding and intense menstrual pain [7,8]. Menstrual pain is also influenced by uterine position, with higher pain intensity being associated with a marked flexion of the corpus on the cervix, particularly with an angle above 210° [9].

The aim of this study is to assess whether a marked flexion of the uterine corpus on the cervix can also be related to the presence of adenomyosis.

## 2. Materials and Methods

This is a single-center prospective observational study performed on premenopausal women referred to our Endometriosis and Chronic Pelvic Pain outpatient clinic at San Martino University Hospital, Genova, between October 2020 and June 2021. All patients in our clinic routinely sign an informed consent to the anonymous use of clinical data. The local ethical committee approved anonymous publication of the data (CER Liguria N773/2021). At consultation, clinical data were collected and each woman was requested to fill a self-administered questionnaire evaluating the presence and intensity of pain symptoms. Intensity of pain was evaluated by a 10 cm visual analogue scale (VAS). Thereafter, each woman underwent abdominal and vaginal examination and transvaginal ultrasound (TVUS) for diagnostic purposes.

Ultrasound investigations were performed by an experienced sonographer using a GE Voluson E6 (GE Medical Systems, Zipf, Austria) ultrasound machine and a wideband of 5–9 MHz transducer. Power Doppler was set to a pulse repetition frequency of 6 kHz to distinguish myometrial cyst from blood vessels. Women were asked to proceed to ultrasound with an empty bladder. Two-dimensional (2D) gray-scale and three-dimensional (3D) ultrasound volumes were performed and saved.

MUSA criteria were used for the diagnosis of adenomyosis [10,11]. Presence of at least two or more of the following features were necessary for the diagnosis: heterogeneous myometrial echotexture; asymmetrical thickening of myometrium; hyperechoic islands and echogenic sub-endometrial lines; hypoechoic striation and fan-shaped shadowing; myometrial anechoic lacunae or cysts (seen as a round anechoic areas within the myometrium); trans-lesion vascularity; globular uterine configuration and/or increased uterine volume; and the presence of a poorly defined, thickened, irregular, and interrupted endometrial-myometrial junctional zone. Patients were excluded from the study when the angle of uterine flexion was possibly distorted by pathologies such as the presence of large myomas or adhesions within adjacent organs (i.e., “question mark sign”), or adhesions documented by no sliding sign of the uterus with adjacent structures. Accuracy of ultrasonography in documenting pelvic adhesions exceeds 95% [12]. Women with endometriosis, ovarian cysts or small fibromas not affecting the uterine angle of flexion were included.

Uterine volume was automatically calculated by the ultrasound machine based on the formula: longitudinal (mm) × antero-posterior (mm) × transverse (mm) diameter × 0.5223.

**Figure 1:**
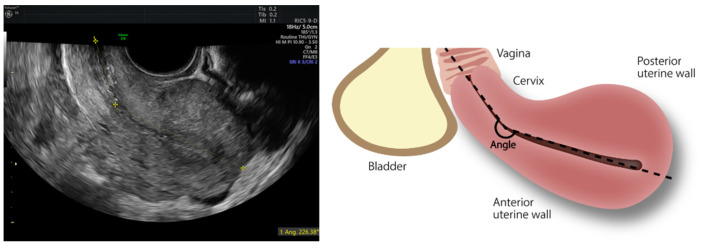
Ultrasonographic and schematic representation of angle of uterine flexion measurement.

Angle of uterine flexion was evaluated as the anterior angle between the longitudinal axis of the cervix and the longitudinal axis of uterine corpus (Figure 1) and was categorized as <150°, between 150° and 210° and >210°, in accordance with a previous investigation where this categorization reflected different intensities of menstrual pain [13].

Ultrasound scans were performed by expert operators.

Statistical analysis was performed by the StatìView 5.1 (SAS Institute, Cary, NC, USA) statistical program. Means were compared by the Student’s t-test. Percentages were compared by the chi-squared test. Single and multiple logistic regression analysis were used to test factors linked to the presence of adenomyosis. A *p* value < 0.05 was considered statistically significant.

## 3. Results

A total of 120 women were included in the study. Two women with large myomas of the uterus were excluded because of the difficulties in defining an accurate angle of uterine flexion. In all other cases, the myomas did not exceed 4 cm in diameter. Another four women with posterior pelvic adhesions were also excluded. Among the included women, ultrasound features of adenomyosis were found in 76 of cases (63.3%). Adenomyosis was classified as either focal (*n* =1) or diffuse (*n* = 75). Among the latter, 46 patients presented diffuse adenomyosis in the whole uterine corpus; 14 women were affected by diffuse anterior wall adenomyosis and 15 by diffuse posterior wall adenomyosis.

The mean age was 35.5 ± 6.8 years and 37.4 ± 6.7 years (*p* = 0.14) in women with and without adenomyosis, respectively. There was no significant difference between the two groups, with the exception of intermenstrual pain that was more intense in women with adenomyosis (4.04 ± 3.79 vs. 2.57 ± 3.34; *p* = 0.034) (Table 1). Uterine volume was significantly higher in women with adenomyosis (*p* = 0.043; Table 1).

**Figure 2:**
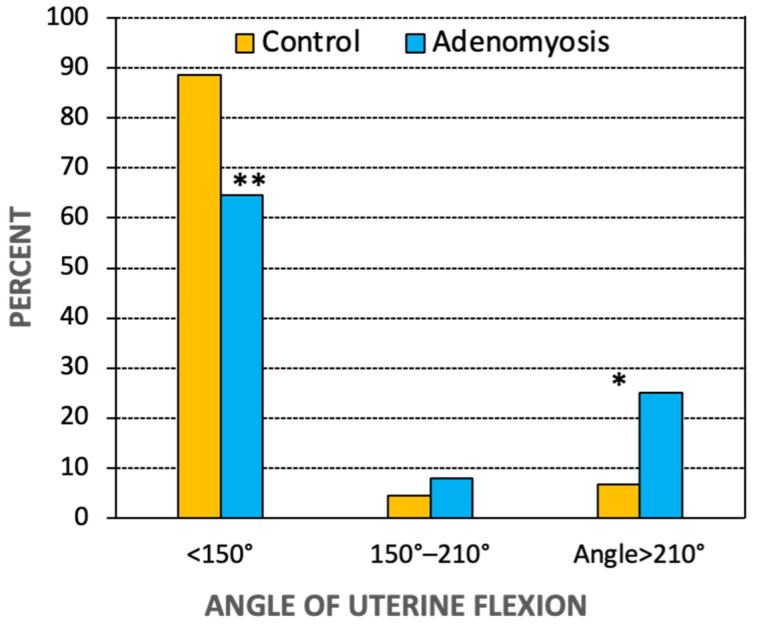
Data on prevalence of adenomyosis stratified by the angle uterine flexion in women without and with a diagnosis of adenomyosis. * *p* = 0.015; ** *p* = 0.004 vs. control.

An angle of uterine flexion <150° was reported in 90/120 women (75%); an angle between 150° and 210° in 8/120 women (6.7%); and an angle > 210° in 22/120 women (18.3%). The distribution of the three angle categories was different in women with and without adenomyosis (Figure 2). The prevalence of an angle of flexion <150° was lower in women with than without adenomyosis (64.5% and 88.6%; *p* = 0.004). The prevalence of an angle between 150° and 210° was similar (7.9% vs. 4.5%; *p* = 0.44), and the prevalence of an angle of uterine flexion >210° was higher in women with than without adenomyosis (25.0% vs. 6.8%; *p* = 0.015).

The univariate logistic regression analysis showed that the presence of adenomyosis was not related to age; BMI; parity; abortions; age at single center prospective observational study; presence of heavy menstrual bleeding, of pain during menses or at intercourse; the use of hormone therapies; or the presence of endometriosis or myomas. Factors significantly related to adenomyosis were the angle of uterine flexion >210° (*p* = 0.022), the uterus volume (*p* = 0.050), and the presence of intermenstrual pain (*p* = 0.038) (Table 2). The multiple logistic regression analysis (R_2_ 0.140) showed that adenomyosis was related only to the presence of an angle of uterine flexion >210° (OR 5.80 95%CI 1.19,28.3; *p* = 0.029) (Table 2).

## 4. Discussion

To our knowledge, this is the first study relating the angle of uterine flexion with the presence of adenomyosis. The data indicate that an angle of uterine flexion above 210° represents a major determinant of adenomyosis, by increasing its prevalence by about 6-folds.

It was previously reported that the modification of uterine position can be associated with a higher intensity of pain, particularly when the angle of uterine flexion exceeds 210° [9,14]. Menstrual pain is the consequence of uterine contractions aimed to expel menstrual blood. During contraction, intra-lumen endometrial pressure increases up to 300 mmHg, and marked distortion of the intra-lumen profile can be observed [15,16]. A stiffer inner uterine orifice (IUO) is harder to dilate, and by counteracting menstrual blood expulsion, menstrual pain increases [17]. An increased angle of uterine flexion may also reinforce the “valve” sited at the IUO, causing stronger uterine contractions and a higher intensity of menstrual pain [9]. Fascinating studies have hypothesized that the elevation of intra-lumen pressure combined with excessive stretching of basal endometrial lamina, due to strong myometrial contractions, may favor the leakage of endometrial cells into the myometrium and the growth of adenomyosis [18].

On these bases, it can be hypothesized that an angle of uterine flexion above 210°, may lead to an excessive intra-lumen pressure and favor the development of adenomyosis. Although this is a possibility, prospective studies would be necessary to define whether the angle of uterus flexion is involved in the pathogenesis of adenomyosis, and possibly also to histologically confirm it. Indeed, the possibility that adenomyosis changes the angle of uterine flexion or that an enlarged adenomyotic uterus is simply dislocated posteriorly due to gravity with a secondary modification of the angle of uterine flexion, cannot be excluded. The data showing that many adenomyotic uteri are not retroflexed does not seem to support this hypothesis.

The strength of this study is that it was performed in a single center by expert sonographers. The major drawback is the inability to provide histological confirmation of adenomyosis. Recent data indicate that imaging techniques such as MRI and transvaginal ultrasound have a high sensibility and specificity in detecting uterine adenomyosis [4,6]. The data were obtained in a single center, in women mainly Caucasian and suffering from pelvic pain. Thus, they can be considered preliminary but need to be obtained in other setting and by other investigators. The early recognition of a risky angle of flexion may allow for the utilization of preventive measures, either medical, by reducing the number and intensity of menstruations, or surgical, by trying to change the angle of uterine flexion [19,20,21].

This study indicates that the ultrasound-estimated angle of uterine flexion >210° is related to the presence of uterine adenomyosis. Whether confirmed in additional prospective studies, the angle of uterine flexion may emerge as a possible important, previously unrecognized risk factor for adenomyosis.

## Figures and Tables

**Table 1:** Characteristics of patients included in the study.

	Mean ± SD	N/Tot	No Adenomyosis	Adenomyosis	*p* Value
			(*n* = 44)	(*n* = 76)	
Age (yrs.)	36.7 ± 6.8		35.5 ± 6.8	37.4 ± 6.7	0.139
BMI (kg/m^2^)	22.7 ± 4.6		21.5 ± 9.7	23.3 ± 5.2	0.189
Age at menarche (yrs.)	12.4 ± 1.5		12.6 ± 1.5	12.3 ± 1.5	0.293
Pregnancy		17/120 (14.2%)	4/44 (9.1%)	13/76 (17.1%)	0.227
Abortions		5/120 (4.2%)	2/44 (4.54%)	3/76 (3.94%)	0.956
Menstrual pain (*n*)		90/120 (75%)	36/44 (81.8%)	54/76 (71.0%)	0.190
(VAS)	5.51 ± 3.60		5.81 ± 3.61	5.32 ± 3.63	0.476
Intermenstrual pain (*n*)		60/120 (50%)	17/44 (38.6%)	43/76 (56.6%)	0.058
(VAS)	3.57 ± 3.69		2.57 ± 3.34	4.04 ± 3.79	0.034
Pain at Intercourse (*n*)		68/120 (56.7%)	25/44 (56.8%)	43/76 (56.6%)	0.983
(VAS)	3.75 ± 3.48		3.64 ± 3.64	3.82 ± 3.41	0.786
Heavy Menstrual Periods		21/120 (17.5%)	9/44 (20.4%)	12/76 (15.8%)	0.524
Hormone therapy		44/120 (36.7%)	12/44 (27.2%)	32/76 (42.1%)	0.104
Uterine volume (cm^3^)	62.2 ± 37.5		53.0 ± 22.2	67.5 ± 43.1	0.040
Angle of flexion (°)	146.1 ± 50.7		127.2 ± 38.2	157.5 ± 54.1	0.001
<150		90/120 (75.0%)	39/44 (88.6%)	51/76 (64.5%)	0.004
150–210		8/120 (6.7%)	2/44 (4.5%)	6/76 (7.9%)	0.473
>210		22/120 (18.3%)	3/44 (6.8%)	19/76 (25.0%)	0.015
Endometriosis		47/120 (39.2%)	21/44 (47.7%)	26/76 (34.2%)	0.146
Myomas		23/120 (19.2%)	10/44 (22.7%)	13/76 (17.1%)	0.454

**Table 2:** Simple and multiple logistic regression analyses between presence of adenomyosis and related factors. No significant relation was observed with age, BMI, parity, abortion, age at menarche, previous surgery, heavy menstrual bleeding, menstrual pain, pain at intercourse, endometriosis, myomas or hormone therapy.

Simple	Simple Regression			Multiple Regression		
	OR	95% CI	*p*	OR	95% CI	*p*
Angle of Flexion > 210°	4.47	1.23, 16.21	0.022	5.80	1.19, 28.3	0.029
Uterus Volume (cm^3^)	1.01	1.00,1.03	0.050	1.01	0.998, 1.03	0.0814
Intermenstrual pain	2.32	1.05, 5.16	0.038	2.17	0.91, 5.17	0.078

## Data Availability

The data presented in this study are available on request from the corresponding author. The data are not publicly available due to privacy as the Ethical Committee recommends.
